# New Diagnostic Signs in Typhoid Diseases

**Published:** 1844-07-13

**Authors:** 


### NEW DIAGNOSTIC SIGNS IN TYPHOID DISEASES.

M. Ranque states, in the Journal de Medicine et de Chirurgie Pratiques, that the pathognomonic sign of a fever which is likely to prove serious, is a coating like mother-o'-pearl of the gums, between the molar teeth. If, in addition, leeches have been applied to the patient, the bites will assume the color of indigo. This symptom is quite characteristic, and when observed, we may rest assured, that however mild the symptoms may appear, they will ere long assume a very serious aspect. M. Ranque further states, when this pearly tint between the molar teeth disappears under the pressure of the finger, the typhoid symptoms will not be very serious; but if the exudation be thick, not disappearing under pressure, and occupying many of the interstices between the teeth, we may look for a severe and long-continued attack. As the disease advances, the colour of the appearance changes; it becomes black, and sordes appear. The same remark applies to the leech-bites. The deeper the indigo colour, the more serious is the attack likely to prove. If these signs exist at the outset of an attack of pyrexia, they are, according to M. Ranque, infallible symptoms, either of a typhoid fever, or of inflammation accompanied by symptoms of a typhoid character.

The papers give a striking instance, illustrative of the effects of mental emotion on the physical frame. An Irish girl, aged seventeen, who had borne a good character, was lately tried at Strafford for having stolen a gown and petticoat, and was sentenced to seven years' transportation. It is now believed that she merely took the goods to wear on some particular occasion, without any intention of keeping them. She heard the judgment, and remained stupified: in twenty-four hours she was a lunatic, and is now in infirmary, with no hopes of recovery. She was a remarkably handsome girl; but, from the period of her sentence, her health visibly declined, and her hair has actually turned gray.—*London Med. Times.*

